# Extremophile Metal Resistance: Plasmid-Encoded Functions in Streptomyces mirabilis

**DOI:** 10.1128/aem.00085-22

**Published:** 2022-05-23

**Authors:** Hanka Brangsch, Marlene Höller, Thomas Krauβe, Mohammed Waqas, Volker Schroeckh, Axel A. Brakhage, Boyke Bunk, Cathrin Spröer, Jörg Overmann, Erika Kothe

**Affiliations:** a Institute of Microbiology, Faculty of Biosciences, Friedrich Schiller University Jena, Jena, Germany; b Leibniz Institute for Natural Compound Research and Infection Biology- Hans Knöll Institute, Jena, Germany; c Leibniz Institute DSMZ-German Collection of Microorganisms and Cell Cultures, Braunschweig, Germany; University of Michigan-Ann Arbor

**Keywords:** *Streptomyces*, heavy metal resistance, cross-kingdom transformation, genome sequence, metal efflux, soil

## Abstract

The extreme metal tolerance of up to 130 mM NiSO_4_ in Streptomyces mirabilis P16B-1 was investigated. Genome sequencing revealed the presence of a large linear plasmid, pI. To identify plasmid-encoded determinants of metal resistance, a newly established transformation system was used to characterize the predicted plasmid-encoded loci *nreB, hoxN*, and *copYZ*. Reintroduction into the plasmid-cured S. mirabilis ΔpI confirmed that the predicted metal transporter gene *nreB* constitutes a nickel resistance factor, which was further supported by its heterologous expression in Escherichia coli. In contrast, the predicted nickel exporter gene *hoxN* decreased nickel tolerance, while copper tolerance was enhanced. The predicted copper-dependent transcriptional regulator gene *copY* did not induce tolerance toward either metal. Since genes for transfer were identified on the plasmid, its conjugational transfer to the metal-sensitive Streptomyces lividans TK24 was checked. This resulted in acquired tolerance toward 30 mM nickel and additionally increased the tolerance toward copper and cobalt, while oxidative stress tolerance remained unchanged. Intracellular nickel concentrations decreased in the transconjugant strain. The high extracellular nickel concentrations allowed for biomineralization. Plasmid transfer could also be confirmed into the co-occurring actinomycete *Kribbella* spp. in soil microcosms.

**IMPORTANCE** Living in extremely metal-rich environments requires specific adaptations, and often, specific metal tolerance genes are encoded on a transferable plasmid. Here, Streptomyces mirabilis P16B-1, isolated from a former mining area and able to grow with up to 130 mM NiSO_4_, was investigated. The bacterial chromosome, as well as a giant plasmid, was sequenced. The plasmid-borne gene *nreB* was confirmed to confer metal resistance. A newly established transformation system allowed us to construct a plasmid-cured S. mirabilis as well as an *nreB*-rescued strain in addition to confirming *nreB* encoding nickel resistance if heterologously expressed in E. coli. The potential of intra- and interspecific plasmid transfer, together with the presence of metal resistance factors on that plasmid, underlines the importance of plasmids for transfer of resistance factors within a bacterial soil community.

## INTRODUCTION

Actinobacteria, in particular, *Streptomyces* spp., are members of soil communities that can be found at highly contaminated sites (1, 2). These Gram-positive bacteria are mostly recognized for their vast metabolic capacity that makes them interesting, among other fields, for bioremediation of soil (3). Streptomyces mirabilis P16B-1 had been isolated from a former uranium mining site, which features high ambient concentrations of metal ions in the soils (2, 4). This strain shows extreme tolerance and grows on media containing 130 mM Ni^2+^ or 5 mM Cu^2+^.

To investigate the extreme metal tolerance of S. mirabilis P16B-1, we obtained its full genome sequence. The mostly linear streptomycete chromosomes constitute 8 to 10 million base pairs, with a high GC content, and code for over 7,000 genes, half of which are unique to the corresponding species (5–7). Circular or linear plasmids have been reported regularly, with many of them being self-transmissible during direct cell-cell contact (8–11). Plasmid-encoded functions have been described, associated with growth under heavy metal and antibiotic stress (12, 13). Indeed, a large linear plasmid had been proposed for S. mirabilis P16B-1 based on pulsed-field gel electrophoresis (2).

From the sequence of the large plasmid, three loci potentially involved in metal resistance were identified and subsequently characterized. Additionally, interphylum conjugation with an Escherichia coli donor was performed using a well-established method that makes use of the machinery for circular plasmid translocation and circumvents the methylation-dependent restriction system by utilizing a nonmethylating E. coli (14–18).

One of the identified metal resistance genes, *nreB*, codes for a major facilitator family (MFS) Ni^2+^/H^+^ antiporter. Many MFS proteins display a 12-transmembrane-domain topology and function as uniporter, symporter, or antiporter for different substrates (19), first identified from a Gram-negative strain, Cupriavidus metallidurans 31A (20). There, NreB was shown to be involved in low-level nickel resistance. Homologs like Klebsiella oxytoca NirA, Hafnia alvei NcrA, or *Synechocystis* sp. strain NrsD have been investigated, and transcription of all these transporters was found to be induced by Ni^2+^ and, in some cases, by Co^2+^ (21–23). Some of these species have been shown to carry *nre*-like Ni^2+^ resistance determinants on transferrable plasmids (20, 24–26). Stoppel and Schlegel (27) screened strains from sites with high Ni^2+^ concentrations and observed that *nre* orthologs were very abundant (20, 28). This transporter belongs to the MFS family, functioning as Ni^2+^/H^+^ antiporter (21–23).

While NreB had been identified as a nickel exporter, a high-affinity nickel uptake system, HoxN, was reported for Cupriavidus necator H16 (29). This member of the NiCoT superfamily of integral membrane transporters can be found in all domains of life (30, 31). Homologs from Helicobacter pylori (NixA) (32), Schizosaccharomyces pombe (Nic1p) (30), Rhodococcus rhodochrous (NhlF) (33), Bradyrhizobium japonicum (HupN) (34), or *Bacillus* spp. (UreH) (35) were found to be specific for Ni^2+^ or allowed transport of either preferred Co^2+^ or Ni^2+^ (36, 37). These transporters show a characteristic eight-transmembrane-domain topology and an energy-dependent high-affinity, low-capacity transport mode (31, 37).

Copper, like nickel, is an essential micronutrient needed as a structural element and for redox reactions, e.g., during respiration and electron transport. Its ability to cycle between the oxidation states Cu(I) and Cu(II) makes it an important cofactor in cuproenzymes, like laccases, superoxide dismutases, cytochrome *c* oxidases, or tyrosinases, while nickel is essential for, e.g., urease. Many cuproproteins are further involved in the homeostasis and storage of the element itself (38, 39). In *Streptomyces* spp., copper was found to be essential for the morphologic development, especially during the transition from vegetative to reproductive growth, where it probably serves as the developmental switch between growth stages (40, 41). For copper homeostasis, the *cop* complex was described, e.g., in Enterococcus hirae (42). The *copYZAB* operon encodes two copper P_1B_-type ATPases for efflux of the metal (CopA, CopB), a copper-responsive repressor (CopY), and a copper chaperone, CopZ (38, 42–44), involved in Cu(I) introduction into the transcriptional repressor CopY (45) that, in turn, controls transcription of *copA* and *copB* in E. hirae (42, 43).

Here, we used the highly metal-resistant S. mirabilis P16B-1, which contains a large linear plasmid, to investigate metal resistance genes. Native plasmid transfer was tested in a microcosm experiment to evaluate the potential of heterologous gene transfer in soil and, hence, allow co-occurring soil bacteria to sustain high metal concentrations.

## RESULTS

### The genome sequencing revealed a large linear plasmid in S. mirabilis P16B-1.

The genome of the highly metal-resistant S. mirabilis P16B-1 comprised a linear chromosome of 8.9 Mbp in three contigs (GenBank accession no. CP074102). Owing to colinearity with other *Streptomyces* genomes and taking into account that genes for plasmid maintenance were not identified, the two additional contigs most likely are parts of the genuine chromosomal sequence, although we cannot rule out that a recent recombination event led to separate fragments in S. mirabilis P16B-1. The genomic GC content of 70.9% and the high number of 8,629 genes are in line with the features observed with the genus *Streptomyces*. Of the 8,629 genes, 8,145 are protein encoding (including 26 predicted secondary metabolite biosynthesis gene clusters), while 97 encode RNAs (6 each for 5S/16S/23S rRNAs, 76 tRNAs, 3 noncoding RNAs [ncRNAs], and 387 pseudogenes).

A separate (linear) contig was annotated as likely plasmid sequence, pI, with 532 kbp (GenBank accession no. CP074103). This sequence features 70.7% GC and encodes 1,313 genes. Using a tool for prediction of secondary metabolite biosynthesis clusters, two orthologs to the dutomycin and tetrocarcin polyketide synthesis pathways were identified. The plasmid exhibited an increased coverage of 350× in contrast to the chromosomal coverage of 125×. Thus, a copy number higher than the chromosome seems possible in the hyphae of S. mirabilis P16B-1.

### Plasmid pI harbors metal resistance genes.

**FIG 1:**
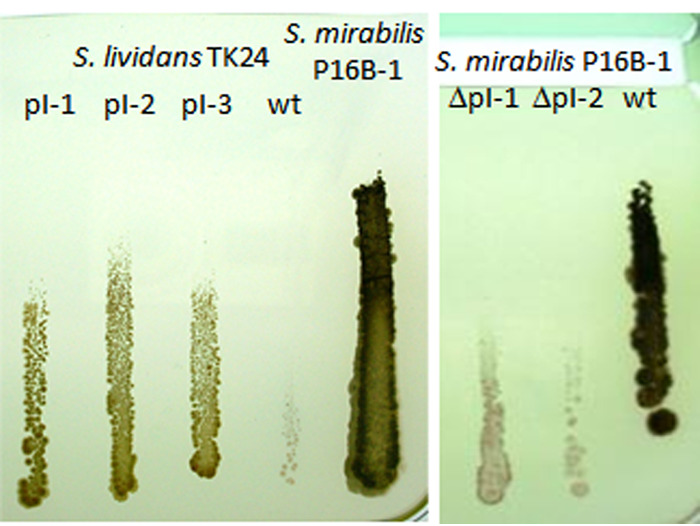
Examples for trench plate tests (TSB; *n* > 3) showing increased tolerance toward Ni^2+^ caused by transfer of plasmid. The first four lines compared three S. lividans transconjugants (T1 to T3) carrying the plasmid to the recipient S. lividans TK24 wild type (WT). For control, nickel tolerance of the donor S. mirabilis P16B-1 (fifth lane) is shown (left). The growth of two cured strains of the donor was added and compared to the S. mirabilis P16B-1 wild type (right). Growth toward a trench containing NiSO_4_ at the top that produced a metal concentration gradient starting from the trench was tested; sensitivity is seen by lack of growth toward the trench. The strong effect on nickel tolerance led to further investigations of nickel resistance.

To test for metal resistance genes encoded on the plasmid, a cured strain of the donor, S. mirabilis ΔpI, that no longer contains the plasmid was used. This cured strain was isolated by exerting temperature stress that is known to lead to loss of plasmids (46). In this case, the strain was plated as described for conjugation, with spores grown in complete medium and exposed to 10 min heat shock at 50°C, followed by 10 min of cooling on ice, before plating on MgCl_2_ and nalidixic acid. This treatment was sufficient to yield a cured strain, S. mirabilis ΔpI. As it was assumed that the plasmid carried determinants for heavy metal resistance, the cured S. mirabilis ΔpI was tested for resistance against nickel, which is tolerated up to concentrations of 130 mM by S. mirabilis P16B-1 (Fig. 1). Indeed, this cured strain did not grow on plates containing more than 13 mM nickel. For comparison, copper and cobalt were also tested in the complex media tryptic soy broth (TSB) and glucose yeast medium (GYM), as well as in minimal medium (AM), to examine the impact of media composition and minimize effects caused by complexation of metals. Cobalt concentrations tolerated were only slightly affected, while copper tolerance was almost not affected by plasmid loss (Table 1).

**TABLE 1:** Maximum metal concentration tolerated depending on the plasmid and sensitivity against oxidative stress (ppm) after 4 weeks of growth in liquid media with different metal concentrations (*n* = 3)^*a*^

Strain	Maximum concn (mM) of NiSO_4_ in:			Maximum concn (mM) of CoSO_4_ in:		Maximum concn (mM) of CuSO_4_ in:		H_2_O_2_ (ppm) in TSB
	TSB	GYM	AM	TSB	GYM	TSB	GYM	
S. mirabilis P16B-1	35	12.5	45	5	1.2	11	2.5	42
S. mirabilis ΔpI	9	1	13	2.5	0.8	11	2.5	30
S. lividans TK24	5	0.8	0.75	3	1	9	2.5	30
S. lividans TK24 pI	25	4.5	10	5	1.4	10	2.5	12

a For control, tolerance of the donor S. mirabilis P16B-1 is shown compared to the cured strain of the donor. After plasmid transfer into S. lividans TK24, the resulting transconjugant (S. lividans TK24 pI) showed gain of metal resistance and loss of oxidative stress tolerance.

### Plasmid transfer to Streptomyces lividans increases metal tolerance.

In addition, the plasmid was transferred to the metal-sensitive model streptomycete S. lividans by conjugation. The heavy metal-sensitive S. lividans TK24 was chosen as recipient since that plasmid-free strain is chloramphenicol resistant, allowing for selection against the chloramphenicol-sensitive donor that carried the plasmid labeled with the apramycin resistance marker (see Fig. S1 in the supplemental material). Interspecific transfer was tested by plate mating on different media. The presence of pI in S. lividans TK24 transconjugants was confirmed by Southern blotting and PCR (Fig. S1 and S2). Three S. lividans transconjugants (T1, T2, and T3; see Fig. S2) harboring plasmid pI were chosen for detailed physiological and morphological characterization. S. lividans TK24 transconjugants harboring plasmid pI showed improved nickel resistance in trench plate tests (see Fig. 1; different media are shown in Fig. S3). In these plates, the metal solution diffuses from a trench cut into the agar medium at the top. Hence, lower to no salts are present in the bottom part of the plate, allowing for comparison with normal, unchallenged growth. The transconjugant growth still was outperformed by the metal-resistant isolate S. mirabilis P16B-1, which suggested the presence of additional resistance determinants on the donor chromosome. An intermediate level of resistance for the S. mirabilis ΔpI strain lacking the plasmid supported that notion.

Growth tests in liquid medium amended with NiSO_4_ showed that the S. lividans TK24 wild type was impaired by 0.75 to 5 mM NiSO_4_, depending on the medium type, while the plasmid-carrying transconjugants showed an increased tolerance of 10 to 25 mM (see Table 1). Resistance to Co^2+^ was also influenced in transconjugants, although to a lesser extent. CuSO_4_-amended medium showed some weak changes. Since metal stress and oxidative stress are connected, it was tested whether the plasmid would provide means for increasing the tolerance toward H_2_O_2_. However, in contrast to the increased metal resistance, the plasmid-containing transconjugants tolerated only 12 ppm, less than the S. lividans wild type. Apparently, the expression of pI-encoded proteins led to a higher oxidative stress sensitivity.

### Biomineral formation is enhanced in the presence of the plasmid.

**FIG 2:**
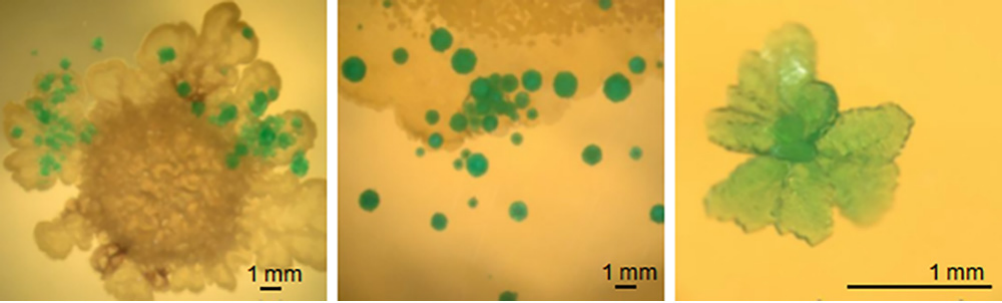
Formation of biominerals below and near colonies of S. lividans TK24 carrying the heterologous plasmid, cultivated on TSB amended with NiSO_4_ at 10 to 20 mM concentrations (for comparison to other strains, see Table S1 in the supplemental material).

The formation of a nickel-containing struvite mineral [Ni-struvite, Ni(NH_4_)(PO_4_)·6 H_2_O] and nickel-hydrogen phosphate in the vicinity of colonies of S. mirabilis P16B-1 had been observed earlier on complex medium amended with Ni^2+^ salt (47). In the present study, the formation of green crystals was observed when the tested strains were cultivated on Ni-containing TSB agar plates. The minerals were formed directly below the colonies or in the vicinity of the bacterial biomass. They exhibited a hemimorphic shape and were mostly rosettes with sizes ranging from 100 μm to 2 mm (Fig. 2). On plates inoculated with the plasmid-bearing transconjugant, biomineral formation that occurred notably increased (Table S1). These both occur directly on, but also in the vicinity of, the strain but not on non-inoculated plates.

### Identification of putative metal resistance genes on the plasmid.

Putative metal resistance genes were identified *in silico*. A putative Ni^2+^ efflux pump (*nreB*), a Ni^2+^ transporter of the NiCoT family (*hoxN*), and two copper resistance determinants (*copZ*, *copY*) were further investigated (Fig. S4). Transcriptome and reverse transcriptase quantitative PCR (RT-qPCR) analyses yielded a strong induction by Ni^2+^ addition for *nreB* (Fig. S5; Table S2). The increased transcription rate was reflected in proteomic peptides for NreB, increasing highly when nickel was added to minimal media (Table S3). Downregulation of *hoxN* transcription was seen with a −6.3-fold change, and repression was down to 0.02-fold for the regulator, *copY*, upon nickel exposure. The chaperone encoding gene *copZ* was upregulated 4.2-fold as a consequence.

As NreB showed a strong response to nickel addition, it was further analyzed. The *nreB* gene (SMIR_42025) with significant similarity to the chromosomal *nreB* of Achromobacter xylosoxidans represented the single locus of this type in S. mirabilis P16B-1; no homologs were detected on the chromosome. In contrast to other NreB-encoding genes, S. mirabilis *nreB* did not appear to be part of an operon (see Fig. S4C). However, the region also codes for the ArsR family transcriptional regulator, KmtR, indicating a potential role in *nreB* transcriptional regulation (Fig. S4C).

NreB (444 amino acids [aa], 46.6 kDa) was identified through BLASTP amino acid alignment as an MFS class H^+^ antiporter (Pfam07690, TIGR00900) with 12 predicted transmembrane domains. The conserved domain search furthermore identified a putative substrate translocation pore. Based on its phylogenetic position (Fig. S6; alignment, Fig. S7), a role in Ni^2+^ and potentially also Co^2+^ tolerance may be predicted.

### Cosmids containing *nreB*, *hoxN*, or *copZY* are involved in metal homeostasis.

**FIG 3:**
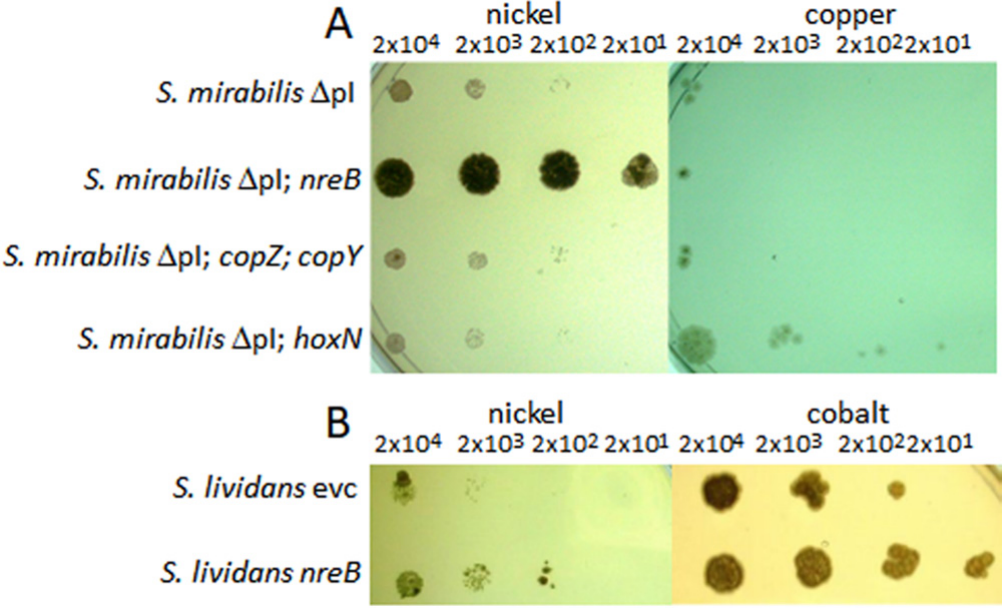
Drop plate test for metal sensitivity of selected strains. (A) GYM medium with 2.5 mM NiSO_4_ (left) or TSB with 8 mM CuSO_4_ (right) to evaluate the potential resistance determinants carried on three cosmids carrying either *nreB* and *kmtR*, *copYZ*, or *hoxN* reintroduced into the cured S. mirabilis ΔpI (for constructs, see Fig. S4 in the supplemental material). (B) TSB with 5 mM NiSO_4_ (left) or 4 mM CoSO_4_ (right) for checking the effect of *nreB* cloned with its native S. mirabilis promoter only, compared to the empty vector control (evc), transfected into S. lividans TK24. The spore counts applied with each drop had been derived from plates with the same agar, but lacking the metal, are given at the top.

Three parts of the plasmid containing the putative resistance genes were used in cosmid construction and introduced into the cured, plasmid-free strain S. mirabilis ΔpI, yielding S. mirabilis ΔpI-*nreB*, S. mirabilis ΔpI-*copZ-copY*, and S. mirabilis ΔpI-*hoxN*. In a drop plate test, metal tolerance was evaluated (Fig. 3A). The fragment containing *nreB* strongly increased the resistance toward Ni^2+^. In contrast, the *hoxN* (SMIR_41220)-containing cosmid had a slightly negative effect on Ni^2+^ resistance. However, this strain showed better growth on TSB amended with CuSO_4_. The genes described for copper resistance, *copY* and *copZ* (SMIR_41155 and SMIR_41160, respectively), affected neither Ni^2+^ nor Cu^2+^ resistance under the tested conditions. Since *nreB* showed the strongest effects on metal resistance, it was further characterized. Strong differences induced by nickel were found, indicating an impact of nickel on both S. mirabilis and S. lividans with 1,597- and 12,837-fold-higher transcript accumulation when metal was added (compare in Table S2). Compared with that of the reference genes, expression in S. lividans was at least comparable to that in S. mirabilis P16-B1 (compare in Fig. S5).

### *nreB* increases nickel (and cobalt) resistance.

To analyze the gene function, the gene was deleted by targeted gene replacement, yielding S. mirabilis Δ*nreB*. Ni^2+^ resistance was impaired, with a drop from 35 to 17.5 mM NiSO_4_ being tolerated on complex media (Table 2). With Co^2+^, a slight loss in tolerance was observed, while complementation rescued metal resistance. An associated phenotype was observed with the deletion of *nreB* in increased sensitivity toward oxidative stress. While the wild type tolerated 42 ppm H_2_O_2_ in TSB liquid medium, the deletion strain only grew to two-thirds of that, with concentrations of 30 ppm (Table S4).

**TABLE 2:** Maximum concentrations tolerated after 4 weeks of growth in liquid cultures (*n* = 3)^*a*^

Strain	Maximum concn (mM) of NiSO_4_ in:			Maximum concn (mM) of CoSO_4_ in:		Maximum concn (mM) of CuSO_4_ in:	
	TSB	GYM	AM	TSB	GYM	TSB	GYM
S. mirabilis P16-B1 strains							
Wild type	35	12.5	45	5	1.2	11	2.5
pI-Δ*nreB*	17.5	6	27.5	2	0.6	11	2.5
pI-Δ*nreB*+*nreB*	25	10	47.5	4	1	ND	ND
Second copy of *nreB*	40	15	45	4	1	11	2.5
S. lividans TK24 strains							
Wild type	5	0.8	0.75	3	1	9	2.5
pI	25	4.5	10	5	1.4	10	2.5
+*nreB*+*kmtR*	20	3	5	5	1,8	9	2.5
pI-Δ*nreB*	20	3.5	2.5	4	1.2	ND	ND

a The metal-resistant S. mirabilis wild type carrying pI and the cured strain retransfected with a pI copy where *nreB* had been deleted (pI-*nreB*), the *nreB* retransformation (+*nreB*), as well as integration of a second copy of *nreB* into the genome were analyzed, while the metal-sensitive S. lividans was transfected with the plasmid carrying metal tolerance genes (pI), a plasmid pI copy that was deleted for *nreB*, and a cosmid containing only the part of the plasmid that carries *nreB* as well as the putative regulator gene *kmtR*. ND, not determined.

Introduction of a second copy in the wild-type S. mirabilis P16B-1 slightly increased Ni^2+^ resistance in liquid culture (compare Table 2). Since a regulation of NreB expression could be the reason for the lack of a stronger effect, the transcriptional regulator KmtR encoded upstream of *nreB* was tested for an influence on nickel resistance. When transformed together with the transporter gene into the plasmid cured S. mirabilis ΔpI, the resulting *nreB*- and *kmtR*-carrying cotransformants showed a marked increase in Ni^2+^ resistance (see Fig. 3A).

### Heterologous expression of *nreB*.

For testing the function of NreB independently from a specifically metal-adapted background, the transporter was heterologously expressed in the Ni^2+^-sensitive model strain S. lividans TK24. The 3,400-bp construct carrying *nreB* and *kmtR* increased nickel tolerance to at least three times the concentration in the recipient strain from 5 to 20 mM NiSO_4_. At the same time, Co^2+^ tolerance was only slightly increased (see Table 2).

To further test whether additional genes on the large plasmid of S. mirabilis P16B-1 are involved in the extremely high nickel resistance, the plasmid and a plasmid carrying the *nreB* deletion, pIΔnreB, were separately transferred to S. lividans TK24 by conjugation. Again, deletion of *nreB* decreased Ni^2+^ resistance compared to the complete plasmid pI (compare in Table 2), while Co^2+^ tolerance was only slightly decreased, and Cu^+^ resistance remained unaffected. Taken together, NreB is responsible for a large part, but not all of, the nickel resistance phenotype encoded on pI, as the S. lividans TK24 strain carrying pI with *nreB* deletion still tolerated higher metal concentrations than the wild type (see Table 2).

In addition, a construct with the native promoter was tested in the heterologous host. Integration again increased Ni^2+^ and Co^2+^ resistance in S. lividans more than 5-fold, showing that the promoter also could be activated despite the missing transcriptional regulator KmtR (Fig. 3B).

**FIG 4:**
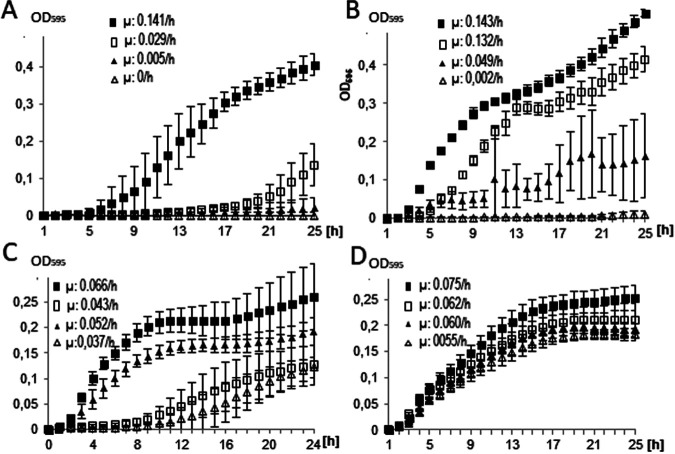
Growth on metal-containing media of E. coli overexpressing *nreB*. The cells carried either *nreB* cloned in-frame (black squares or triangles) or the empty vector control pTrc99a (open squares or triangles). E. coli was grown in LB medium supplemented with NiSO_4_ at 2 mM (squares) and 2.5 mM (triangles) concentrations (A), NiCl_2_ at 1.5 mM (squares) and 2 mM (triangles) (B), CoSO_4_ at 0.5 mM (squares) and 1 mM (triangles) concentrations (C), and CuSO_4_ at 2.5 mM (squares) and 3 mM (triangles) concentrations (D); bars indicate standard deviation of six replicates. The growth rates μ [h] are indicated in each diagram for every growth curve.

To determine the substrate spectrum of NreB and whether the transporter could also work in more distantly related bacteria, the gene was expressed in E. coli, which increased nickel resistance to 2.5 mM NiSO_4_ and 2 mM NiCl_2_, where the empty vector control did not grow (Fig. 4). NreB expression improved growth only slightly on cobalt and did not visibly affect growth with Cu^+^. Statistical analysis of growth rates revealed significant differences between E. coli strains carrying *nreB* and the control (*n* = 6, *P* = 0.05; Table S5). The increase in nickel and partly cobalt tolerance also with a proteobacterial host upon transformation with a construct that contained *nreB* under the control of a suitable promoter in the E. coli expression vector pTrc99a indicates that NreB could be considered to be mainly transporting Ni^2+^ (see Fig. S8 for cloning and expression in E. coli).

We also tested whether NreB could be expressed from its native streptomycete promoter in E. coli. Promoter recognition in more distantly related species would be a prerequisite for conferring resistance to a larger variety of members of a natural community after transfer of the plasmid. An enhanced growth of E. coli was scored on nickel-containing medium even with this construct carrying both *nreB* and *ktmR* (Fig. S9; compare in Fig. 4 for constructs).

### Conjugational transfer genes predicted *in silico*.

It had been shown that transfer into S. lividans was possible. Hence, homologs to genes encoding known transfer proteins from other *Streptomycetes* were searched for *in silico*. Three genomic loci were identified encoding conjugational transfer proteins. One, locus tag SMIR_04920, coded for a predicted TraG protein of the TraG-D_C family (Pfam 12696), while SMIR_12615 and plasmidal SMIR_41765 encoded homologs of the mycobacterial conjugational transfer ATPase MT3759 of the CpaF family. Furthermore, three genes for a type IV secretion system for DNA transfer were found, VirD4, TraC, and VirB4 (SMIR_19795, SMIR_19800, and SMIR_19805, respectively). The gene SMIR_39675 (Pfam01580) had significant similarity to genuine TraB proteins and was located on the chromosome within one smaller contig.

**FIG 5:**
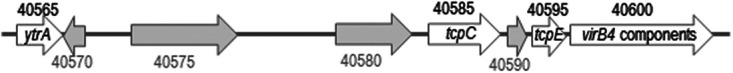
Genomic region for potential plasmid transfer functions. Plasmid loci SMIR_40565 to SMIR_40600 encoding putative plasmid transfer proteins *vtrA*, *tcpC*, *tcpE*, and *virE* components are shown. ORFs without predicted function are shaded in gray. Accession numbers are given for gene identification.

On the large plasmid, none of the predicted proteins could be identified as TraB homologs. However, three loci encoded proteins which could be part of an operon for a plasmid transfer machinery (Fig. 5). SMIR_40600 showed 54% similarity to the conjugative protein SCP1.146 in S. coelicolor A(3)2. A conserved domain search classified the protein as ATP/GTP binding with an AAA-like domain (Pfam12846). SMIR_40585 encoded a predicted conjugative transposon protein, TcpC (pfam12642), while SMIR_40565 encoded a transcriptional repressor that showed 30% homology to TraR of *Streptomyces* sp. strain 14R-10. A UvsW-like helicase, TtrA, was identified (SMIR_42860), showing 33% identity to the S. lividans protein (GenPept accession no. AAO61192). Like in S. lividans, the coding gene was located terminally on the linear plasmid arm. A homolog to a terminal maintenance protein, Tpg, was present on pI upstream of the TtrA-encoding gene. This open reading frame (ORF), SMIR_42855, exhibited 88.5% identity to the terminal protein TpgA1 of *Streptomyces* sp. strain WAC02707 (GenPept accession no. WP_125774744) and 41.1% identity to the plasmid-encoded Tpg protein of Streptomyces clavuligerus ATCC 27064 (GenPept accession no. EFG04331).

This analysis confirmed that the components for plasmid pI transfer that had been achieved, S. mirabilis P16B-1 to S. lividans TK24, are indeed encoded on the plasmid. We therefore wanted to test whether a plasmid transfer in a more natural soil microcosm setting was possible.

### Intragenus plasmid transfer in a native soil microcosm setting.

In order to test if plasmid transfer could be of ecological significance in natural systems, the conjugation experiments were repeated in sterile soil microcosms. Two types of soil were chosen, control soil from a municipal park in Jena (Germany) that was low in heavy metal content, as well as soil from the acidic, highly heavy metal-contaminated area where S. mirabilis P16B-1 originally had been isolated. It was expected that both strains survive in the park soil, while only S. mirabilis P16-B1 would survive in the high-metal soil (47). This setup was used to determine whether metal stress would promote plasmid transfer under natural conditions. Transconjugants could be isolated exclusively from Jena park soil, while the metal-rich soil did not yield transconjugants. A concomitant transfer of a second plasmid (potentially one of the single contigs in sequencing) could neither be seen on a plate nor in microcosms (compare in Fig. S2).

### Plasmid transfer to co-occurring soil bacteria.

**FIG 6:**
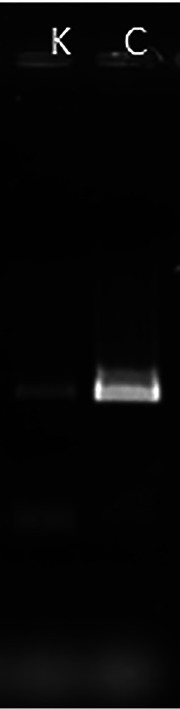
Detection of plasmid transfer from S. mirabilis P16B-1 to *Kribbella* spp. Potential *Kribbella* transconjugant DNA was checked by PCR (K) and compared to S. mirabilis P16B-1 DNA (C) as a positive control. For detection of plasmid transfer, primers were designed that target plasmid sequences and do not match sequences elsewhere in known genomes (compare in Table 3). Negative controls for *Kribbella* before plasmid transfer were performed (data not shown).

Potential transfer of the plasmid to co-occurring isolates obtained from the site where the metal-contaminated soil had been sampled and where S. mirabilis P16B-1 had originally been isolated was tested. The isolates were identified through 16S rRNA gene sequencing. The isolates were chosen to cover a wide variety of bacterial kingdoms, with Gram stain-positive Actinobacteria (the genera *Kribbella*, *Oerskovia*) and Firmicutes (*Bacillus*, *Virgibacillus*), as well as Gram-negative kingdoms Proteobacteria (Pseudomonas, *Stenotrophomonas*) and Bacteroidetes (*Sphingobacterium*, *Pedobacter*). While no viable transconjugants could be obtained from most of these strains, the transfer of pI was successful for the actinobacterium *Kribbella* spp. (Fig. 6), as proven by PCR targeting the selectable marker gene. However, the plasmid was not maintained over a prolonged period of time in *Kribbella.*

## DISCUSSION

Gene flux involving *Streptomyces* has mostly been reported with other actinobacteria (48, 49). Little is known about the potential transfer to more distantly related species, as genus- or species-specific barriers may inhibit successful expression (50). Even promiscuous plasmids, e.g., those of the incompatibility group IncP-1, have limits to their host range (51). However, the presence of stressors like antibiotics or metals can modulate the plasmid transfer frequency and avoid loss of genetic elements within a community (1). With this investigation, we show that extremophilic streptomycetes can harbor mobile genetic elements that enable actinobacterial community members to cope with high metal concentrations found in former mining areas, i.e., 110 ppm nickel and >160 ppm copper (4). The large plasmid of S. mirabilis P16-B1 thus can be used as a model to study linear plasmid transfer in a community and the resultant accelerated adaptation of streptomycetes (and potentially also other actinobacteria) to metal stress in the environment.

In the new host, pI conferred resistance to nickel, copper, and also cobalt. At the same time, the level of resistance did not reach the extreme values that are seen with S. mirabilis P16-B1, a strain that has been isolated from the aforementioned former uranium mining site (2). While the plasmid carries heavy metal resistance determinants and may distribute them to other actinobacteria present in metal-contaminated soil, additional determinants of heavy metal resistance to those encoded by the plasmid must be responsible for the extremely high nickel resistance in S. mirabilis P16-B1. This is specifically visible with the strain that lacked the plasmid and still showed intermediate metal resistance.

Although transfer to another actinobacterium, *Kribbella*, was observed, the plasmid could not be stably maintained in the latter host. Therefore, mycelial distribution and maintenance of pI seem to be dependent on host factors, while genes for conjugation apparently are plasmid derived.

An *in silico* search for transfer determinants identified a locus encoding the conjugative transposon protein TcpC, which is involved in conjugational transfer of single-stranded DNA in Clostridium perfringens (52). A TcpC homolog is located on a linear plasmid of S. coelicolor A3(2) as well (53). The plasmid-encoded SMIR_40565 showed homology to TraR, a GntR-type transcriptional repressor controlling *tra* gene transcription (54). Furthermore, a homologue of TtrA, a helicase required for strand displacement (55), was detected on pI. Directly linked, another protein for maintenance and spreading of linear plasmids, Tpg (56), was identified. The two genes *tap* and *tpg* code for a telomere-associated and a terminal end-patching protein in Streptomyces rochei, respectively (57). No (co)transfer of another plasmid was observed, although a genuine *traB* homologue was identified on one contig. Therefore, it is assumed that this is part of the chromosomal information. This is supported by the lack of other functions promoting transfer, spreading, or maintenance in a new host.

Metal resistance mechanisms located on transferrable plasmids have been found in multiple cases. A model for this was shown to be Cupriavidus metallidurans CH34, which was isolated from a tank of a zinc factory (25). Compared to the extreme nickel resistance of up to 130 mM in S. mirabilis P16B-1, the metal tolerance in this and other proteobacteria is only in the low-millimolar range. The extreme resistance, especially toward nickel but also to very high copper and appreciable cobalt concentrations, indicated that other genes encoding specific mechanisms of extreme heavy metal resistance must be present in S. mirabilis P16B-1. In addition to other factors for metal tolerance, the copy number of the plasmid may aid metal adaptation.

Indeed, metal tolerance could be assigned to genes located on the transferable plasmid, which reduced the intracellular nickel concentration in the heterologous *Streptomyces* host under metal stress. Specifically, the nickel efflux pump NreB, as shown by heterologous expression, enabled growth at elevated Ni^2+^ levels. It appeared to be insufficient to obtain the full nickel resistance in the new host, which might also be explained by different copy numbers, depending on plasmid maintenance in the new host, as well as different transcript levels, owing to availability of regulatory components.

Dependent on living bacterial cells, microbially induced mineral formation was observed. For S. mirabilis P16B-1, struvite (a magnesium-containing ammonium phosphate) or a nickel phosphate had been observed to be formed earlier (47). In addition, an ammonium phosphate mineral, nickel struvite, had been described to be formed due to metabolic activity and high local metal concentrations surrounding the cells of other metal-resistant strains isolated in the same former mining area (3, 58). The same morphology and color observed here indicated that in S. mirabilis, Ni-struvite is the precipitated mineral as well. This was found also with the heavy metal-sensitive S. lividans TK24. Thus, the formation of the biomineral Ni-struvite was not specific to S. mirabilis P16B-1 but, rather, is an example of induced biomineralization (46, 59). The valence state of nickel is not changed in these minerals, excluding the involvement of redox reactions to explain the involvement of living cells. The presence of dead (autoclaved) cells did not promote mineral formation under all conditions tested. Hence, the adsorption to the cell wall-promoting mineral nucleation was not the reason for mineralization. The minerals were observed either on the colonies or in their direct vicinity, which seems to indicate excretion or changes in pH to promote for mineralization. In a soil environment, the mineral formation would lead to reduced bioavailability of nickel, which provides a community function allowing less extremophilic organisms to grow.

The capability of transfer and expression of *nre*-type resistance determinants from one genus to another has already been observed (20, 60), ascribing them a broad host range, which was consistent with the results of the present study. Due to this characteristic, the *nre* resistance determinant of C. metallidurans 31A has already been utilized for the construction of minitransposons and successfully applied in transformation of different bacteria, e.g., endophytic *Burkholderia* spp. and *Herbaspirillum* spp. (61, 62). Former studies of NreB homologues showed that their expression is inducible by Ni^2+^ (21, 63), in concordance with this study.

Two other potential metal resistance genes showed less impact on the metal resistance of a receiver streptomycete. Interestingly, the predicted nickel transporter HoxN, rather, was associated with copper tolerance, while the predicted copper resistance genes *copYZ* did not influence metal tolerance to a marked extent. This does not exclude the possibility of an association with other functions encoded on the plasmid that were not cointroduced in our experiment; the genes *copYZ* might still be part of metal homeostasis in S. mirabilis P16B-1.

Expression of the genes with and without nickel for potential induction of the genes was tested, and a more than 15-fold increase of *nreB* transcript was supported with a concomitant increase in the encoded NreB protein levels upon nickel addition to the medium, while *hoxN* and *copY* were repressed; the chaperone-encoding gene *copZ* was again found to be upregulated.

All in all, the finding of a large linear plasmid and its involvement in metal resistance could show that transfer under native conditions may lead to enhanced community adaptation. The transfer and selection pressure by metal availability in the habitat would promote the growth of streptomycetes able to receive the plasmid, aiding those already well-suited soil organisms to survive in environments with heterogenous distribution of stressors like heavy metals due to their filamentous growth and ability to form exospores. This might give an explanation for the observation that specifically streptomycetes show a high prevalence in heavy metal-contaminated soils (64).

## MATERIALS AND METHODS

### Bacterial strains, cultivation, and microcosm setup.

S. mirabilis P16B-1 (2) is an environmental isolate from a former uranium mining site with high concentrations of soluble metal ions in the soil. A derivative lacking endogenous plasmids, S. mirabilis ΔpI, was obtained in this study by applying heat shock. The plasmid loss was monitored by Southern blotting as well as PCR using primers for plasmid-specific genes. The genetic model organism S. lividans TK24 (str^−^, SLP2 SLP3^−^) (65) was used as recipient in conjugation and transformation experiments. The streptomycetes were grown in mannitol soy medium (65), casein starch medium (CSA; 10 g/L starch, 1 g/L casein hydrolysate, and 0.5 g/L K_2_HPO_4_), minimal medium AM (66), tryptic soy broth (Carl Roth, Karlsruhe, Germany), or glucose yeast medium (GYM; DMSZ, Germany). Metal salts were added if indicated, and oxidative stress was exerted by adding H_2_O_2_ of up to 40 ppm depending on growth at lower concentrations.

Escherichia coli DH5α, ET12567 (pUZ8002; *dam*^−^, *dcm*^−^, *hsdS*^−^, cm^r^) (67), BW25113 (68) and TransforMax EC100D pir-116 (Epicentre Biotechnologies, Madison, WI, USA) with plasmids pSET152 (14), pIJ790 and pIJ773 (18), pTrc99A (69), and pKOSI (17) were used for cloning. E. coli was grown in complex media standard I, super optimal broth (SOB), or LB (all by Merck, Darmstadt, Germany).

### Genome sequencing and assembly.

High-molecular-weight DNA was prepared using Qiagen genomic tip 100/G (Qiagen, Hilden, Germany) according to the manufacturer´s instructions. SMRTbel template library was prepared according to the instructions from Pacific Biosciences (Menlo Park, CA, USA). Briefly, for preparation of 15-kb libraries, 8 μg genomic DNA was sheared using g-tubes (Covaris, Woburn, USA) according to the manufacturer’s instructions. DNA was end repaired and ligated overnight to hairpin adapters applying components from the DNA/kit P6 from Pacific Biosciences. Reactions were carried out according to the instructions of the manufacturer. BluePippin size selection to greater than 7 kb was performed according to the manufacturer´s instructions (Sage Science, Beverly, MA, USA). Conditions for annealing of sequencing primers and binding of polymerase to purified SMRTbel template were assessed with the calculator in RS Remote (Pacific Biosciences). Single-molecule real-time (SMRT) sequencing was carried out on the PacBio RS II (Pacific Biosciences), taking a 240-minute movie on a total number of two SMRT cells.

Both PacBio runs yielded 155,230 reads, with a mean read length of 10,521 bp. SMRT cell data were assembled using the RS_HGAP_Assembly.3 protocol included in SMRT Portal v2.3.0 using default parameters. The assembly revealed nine contigs from which four were found to be largely redundant to the bacterial chromosome and thus excluded from genome submission. None of the contigs were found to be circular in terms of redundant overlapping contig ends. Raw genome annotation was carried out using Prokka 1.8 (70).

### Transcriptome and proteome profiling.

To evaluate the impact of nickel on gene expression, RNA sequencing was performed. S. mirabilis P16B-1 was grown in minimal medium (AM) (66) with or without 5 mM NiSO_4_ added. Cells were grown and monitored using a cell growth quantifier (aquila biolabs, Baesweiler, Germany). During the exponential phase, the cells were harvested by filtering the culture through Miracloth (Millipore, Burlington, USA) and frozen in liquid nitrogen. Total RNA was extracted after pulverization with a porcelain mortar and pestle and stored at −70°C if necessary. RNA was isolated using the innuPrep plant RNA kit (Analytik Jena, Jena, Germany). Additionally, on-column DNA digestion was performed with only 300 μL of washing solution HS, centrifugation for 1 min, adding 75 μL of DNase I incubation mix (1.5 μL DNase I and 73.5 μL DNase buffer, Analytik Jena, Jena, Germany), and incubation for 15 min at room temperature. Then, another 300 μL of washing solution was added and the tubes centrifuged again for 1 min. Further washing steps were performed as described by the manufacturer. A second DNase treatment was performed with DNase I set (Zymo Research, Irvine, CA, USA) and stopped with cleaning (RNA Clean & Concentrator-25 kit; Zymo Research, Irvine, USA). The RNA concentration was determined spectrophotometrically, and the transcriptome sequencing (RNA-seq) was performed by StarSeq (Mainz, Germany) with three technical and biological replicates. The data were analyzed for gene identification, and those genes encoded on the plasmid pI were evaluated for statistically relevant changes. Genes with at least 2-fold regulation upon nickel addition to the medium were selected. The genes identified for potential metal homeostasis investigated in this work were among those further analyzed.

For protein abundance measurements (71), cells grown as for transcriptome analyses were harvested and protein concentrations measured (Sigma-Aldrich, Darmstadt, Germany). After digestion using 50 ng/μL trypsin-LysC (mass spectrometry grade; Promega, Mannheim, Germany) in 50 mM NH_4_HCO_3_, peptides were extracted and analyzed as described (71) using liquid chromatography-tandem mass spectrometry (LC-MS/MS) analysis (UltiMate 3000 RSLCnano, QExactive HF; Thermo Fisher Scientific, Waltham, MA, USA) with initial peptide trapping (5 min, Acclaim PepMap 100; 5 μL/min) followed by separation (Acclaim PepMap RSLC nanocolumn). Positively charged ions (2.2 kV on nanospray flex ion source; Thermo Fisher Scientific) were detected on the quadrupole-Orbitrap instrument after LC-MS/MS using Chromeleon 7.2, Q Exactive HF Tune 2.8, and Xcalibur 4.0 software (Thermo Fisher Scientific). At least two peptides per protein and a strict false-discovery rate of <1% (reverse decoy) were required for assignment, and quantification was performed on data normalized using the total peptide amount approach. The data were analyzed for occurrence in both data sets, with and without nickel, and abundance change was determined. Genes with at least 2-fold regulation upon nickel addition to the medium were selected. Only one of the proteins studied here was present, *nreB*, which showed very high abundances in the nickel treatment but was absent when no additional nickel was supplied to the growing cells (as were the genes *kmtR*, *copYZ*, and *hoxN* in all treatments).

### *In silico* analyses.

Similarity searches on the gene and protein levels were performed using BLAST. For proteins, the Swiss-Prot and NCBI RefSeq databases were queried. NCBI conserved domain database and the transporter classification system (72) were consulted for categorization of putative transporter proteins.

Protein sequences were aligned using MAFFT online v7 (BLOSUM62 scoring matrix; gap opening penalty, 1.53; offset value, 0.2) (73) and edited using BioEdit. Maximum-likelihood tree calculation and bootstrapping were executed by raxmlGUI v1.3.1 applying Protgramma rate distribution and Dayhoff amino acid similarity matrix with 200 bootstrap repetitions. Phylogenetic trees were visualized using FigTree v1.4.3 (http://tree.bio.ed.ac.uk/software/figtree) and graphically finalized using CorelDraw 11. Figures displaying sequence alignments was possible using Clustal Omega (https://www.ebi.ac.uk/Tools/msa/clustalo/). AntiSMASH was used for prediction of antibiotic biosynthesis gene clusters encoded on the plasmid (74).

### DNA isolation, PCR, and cloning.

*Streptomyces* species genomic DNA was isolated according to a modified salting-out procedure (75) with an additional hexadecyltrimethylammonium bromide (CTAB) step, whereafter proteinase treatment, 130 μL CTAB (10% cetrimonium bromide dissolved in 0.7 M NaCl) was added to 700 mL reaction volume and incubated for 10 min at 55°C and 5 min at 37°C. This additional step was followed by the chloroform/isoamyl alcohol extraction of the original protocol. DNA concentration and purity were checked spectrophotometrically (DS-11 spectrophotometer; DeNovix, Wilmington, DE, USA).

For cloning, Phusion high-fidelity DNA polymerase (New England Biolabs, Ipswich, UK) with GC buffer supplemented with 1 M betaine and 8% dimethyl sulfoxide (DMSO) or PrimeStar GXL DNA polymerase (TaKaRa Bio, Kusatsu, Japan) was used. PCRs were run in Tprofessional standard thermocycler gradient or T3 thermocycler (Biometra, Göttingen, Germany) using two-step and three-step PCR programs. Primer synthesis (Table 3) and PCR product sequencing were executed by Eurofins Genomics (Ebersberg, Germany).

The PCR products were purified using QIAquick PCR purification kit (Qiagen, Venlo, Netherlands) and digested with appropriate restriction enzymes (New England Biolabs). Ligation into vectors was carried out with T4 ligase (Thermo Fisher Scientific) at 14°C overnight. E. coli transformation was conducted by electroporation, and plasmids were isolated by using GeneJet plasmid miniprep kit (Thermo Fisher Scientific).

RT-qPCR was performed to check transcriptional gene accumulation levels. Cells were grown and monitored using a cell growth quantifier (aquila biolabs, Baesweiler, Germany) in TSB medium, with or without 10 mM NiSO_4_ (*n* = 3). After harvesting and RNA extraction (see above), cDNA synthesis was performed using iScript cDNA synthesis kit (Bio-Rad, Hercules, CA, USA). The RNA concentration was adjusted to 25 ng/μL. For qPCR, 3.13 μL of SYBR green master mix (Thermo Fisher Scientific), 1.53 μL ultrapure water, 0.3 μL 10 mM primers (Table 3), and 1 μL cDNA was used with initial denaturation for 5 min at 95°C, followed by 40 cycles (denaturing, 95°C, 30 s; primer annealing, 60°C, 30 s; elongation, 72°C, 45 s) using qTower3 (Analytik Jena, Jena, Germany). As reference genes, *gyrB*, *rpsA*, and *infB* were applied in addition to *nreB*, using three technical replicates. Relative expression was calculated with qPCRsoft v3.4 with threshold cycle (ΔΔ*CT*) quantification (76), and data were visualized in R using ggplot2 and scales.

**TABLE 3:** Primers used in this study

Primer name	Sequence	Purpose
ApraF	GGTCCACAGCTCCTTCCGTA	Amplification of aac(3)IV fragment
ApraR	TTATGAGCTCAGCCAATCGAC	Amplification of aac(3)IV fragment
P16pII-F	ATGGGTAAGGCGCACTCTGC	Presence of plasmid
P16pII-R	CCCTGGAACTTCGAGACGAGTG	Presence of plasmid
nNreBCon_F	GCGGTACCCCGAAGGTCCCC	Primary cosmid PCR
nNreBCon_R	TCCCCCATCAAGACCCACGG	Primary cosmid PCR
nreBCosm_F	AATTATTCTAGACCCGGCCTCGATCACGCTGC	Secondary cosmid PCR
nreBCosm_R	AATTAACTCGAGGAGAAGGCCCAGGCTCTCGC	Secondary cosmid PCR
nreB-ko_F	GAGGCTCTCCACTGTACCTACATGTGCGCACCTGCGCACATTCCGGGGATCCGTCGACC	Resistance cassette
nreB-ko_R	CCGGCGCGTACGGCACCTCCCCCTCGGCGGCCTGAACCTTGTAGGCTGGAGCTGCTTC	Resistance cassette
nreB-ko-ctr-F	CGTGCCGACCAGGGCGATGA	Deletion control
nreB-ko-ctr-R	GCACCTGACCGGCGTACCGC	Deletion control
nreB-ko-SB-F	CGCTCAGCATCACCACACGG	Southern blotting probe
nreB-ko-SB-R	ACAGTGGCCCTCGGCTTGCT	Southern blotting probe
sodNconF	TTGGTATCATGATGGGACTCGCCTTCCATCTC	Cosmid PCR for plasmid target deletion
sodNconR	TTGGTATCATGAAGTTGAAGATCGTGTCGGGC	Cosmid PCR for plasmid target deletion
K.o.-P16pII-F	GCGTATGGACATCGGTGCGTCATCCTGCCCTGCCGTATGATTCCGGGGATCCGTCGACC	Resistance cassette
K.o.-P16pII-R	GGACGGTGTGGACCAGCGGTGACCGCAAGCGTGGCCTCATGTAGGCTGGAGCTGCTTC	Resistance cassette
K.o.-P16p12-CF	GCCGAACGCTGACCAGCCGC	Deletion control
K.o.-P16p12-CR	CCAGCCTGCCGGACGGTGTG	Deletion control
hphMFS-F	AATTATTCTAGAGGCCCGTCCCGGCGCGTACGTCG	P16nreB for pSEThph
hphMFS-R	AATATTGCGGCCGCTTCACGGCGTGTCTCCACCA	P16nreB for pSEThph
phox-pSET-R	TAGTTTTCTAGACTCAACCGGCGTTTCTGGACC	P16phoxN for pSEThph
phox-pSET-F	AATAATGCGGCCGCGCGGTGCAGGATGTCGGCCAC	P16phoxN for pSEThph
CuP2Cosm-pSET-F	AATATACGCGCGCGATCACGGCCGGGACGAGGAT	copY-copZ carrying fragment of the plasmid for pSEThph
CuP2Cosm-pSET-R	AATATACGCGCGCGGCCGCATGAGCTGGTCACTC	copY-copZ carrying fragment of the plasmid for pSEThph
nreBCosm-pSET-F	AATTACCGCGCGCGTAGACCCGGCCTCGATCACG	P16nreB carrying fragment of plasmid for pSEThph
nreBCosm-pSET-R	AATTACCGCGCGCGTCGCCGAGAACGTAGAGCTC	P16nreB carrying fragment of plasmid for pSEThph
MFS-pTrc-F	AATAAAGGATCCTGCGGTCGGCGGGTCAATTC	P16nreB for pTrc99A
MFS-pTrc-R	AATAAAAAGCTTCCCGCCCGACATCCTCGACC	P16nreB for pTrc99A
nreB-f	CAGGCTCTCCAGGTCATAGG	qRT-PCR primer
nreB-r	GTCCTGATCTTCCTGCTCCAG	qRT-PCR primer
gyrB-f	CGGCCCCACTTGATCTTGTA	qRT-PCR primer
gyrB-r	CGAGAAGCTCCGCTATCACA	qRT-PCR primer
rpsA-f	ACGACCAGGGCAACTACATC	qRT-PCR primer
rpsA-r	CCTCGCGGGACTTGATGAC	qRT-PCR primer
infB-f	GACGCCGAACGGACGAAT	qRT-PCR primer
infB-r	GAACTCGCCAAGGAGTTCGG	qRT-PCR primer

### Conjugation and knockout.

For interspecific conjugation, a protocol developed for streptomycetes (65) was adapted. E. coli ET12567 pUZ8002 cells carrying the desired construct were grown in LB containing chloramphenicol, kanamycin, and vector-specific antibiotics. When an optical density at 600 nm (OD_600_) of 0.6 was reached, the culture was washed with 2× TY (65) and resuspended in 250 μL 2× TY. Streptomycete spores from a 2-week-old plate served as recipient and were resuspended in 250 μL 2× TY with subsequent incubation for 10 min at 50°C and 10 min cooling, E. coli was added, and the mixture plated on MS agar (with 10 mM MgCl_2_) and incubated overnight at 28°C. An overlay (0.9% sodium chloride, 0.6 mg/mL nalidixic acid, and the selective antibiotic) was used to select for streptomyces transconjugants and against the E. coli donor.

For gene deletion, PCR-targeted gene replacement (18) was performed. Cosmids were constructed by PCR amplification of S. mirabilis genomic DNA fragments that were ligated into the cosmid vector pKOSi (17) and introduced in E. coli BW25113 pIJ790. For deletion of the target gene on the cosmid, a resistance cassette was generated by PCR with pIJ773 as the template and transformed into freshly prepared, cosmid-carrying E. coli cells that were grown at 30°C in SOB supplemented with 10 mM l-arabinose to an OD_600_ of 0.6 for competence. Successful transformants and recombinant cosmids were isolated, and a cosmid carrying the gene deletion was used for transformation of E. coli ET12567 pUZ8002, which was subsequently used for conjugation with S. mirabilis. Exconjugants were tested by colony PCR and Southern blot analysis with the digoxigenin (DIG) labeling and detection system (Roche Diagnostics GmbH, Grenzach-Whylen, Germany) to verify a clean knockout.

For genetic complementation of the S. mirabilis deletion strains, the amplified gene of interest was inserted into pSEThph (via XbaI and NotI-HF), transformed into E. coli, and conjugated into S. mirabilis.

### Physiological characterization.

Metal resistance was tested in liquid medium and in 24-well cell culture plates (Cellstar, Greiner Bio-One, Kremsmünster, Austria) using a volume of 2 mL per well. Each well was inoculated with 15 μL of a spore suspension containing 2 × 10^7^ spores/mL. After a first test with metal concentrations increased by 2-fold in each step (*n* = 3), more detailed tests in finer resolution followed.

For trench plate tests, square petri dishes (120 cm by 120 cm; Greiner Bio-One) were filled with medium. A trench of 1 × 10.5 cm was cut at one side of the plate, in which 3 mL of any one of the metal solutions (2 M NiSO_4_, 2 M NiCl_2_, 0.5 M CoSO_4_, or 1 M CuSO_4_) was added. The plates were inoculated with streptomycete spore suspensions and incubated for 4 weeks at room temperature.

In addition to the trench plate test, a drop plate assay was used for testing metal resistance. A four-step dilution series of spore suspension was prepared starting with 10^7^ spores/mL. The suspensions were spotted in 2-μL aliquots and incubated for 1 to 4 weeks at 28°C.

### Determination of biomass metal content.

Streptomycetes were cultivated in liquid GYM amended with 0.25 mM NiSO_4_ in baffled flasks with shaking at 28°C for 5 days, using three biological replicates per strain and treatment. The biomass was obtained and washed twice with distilled water before drying at 40°C and grinding. Metals in the ground biomass were dissolved by microwave digestion using the MarsXpress system (CEM, Kamp-Lintfort, Germany) and measured by inductively coupled plasma-MS (ICP-MS), using a quadrupole ICP-MS spectrometer, XSeries II (Thermo Scientific, Bremen, Germany) in three technical replicates.

### Interspecies plasmid transfer between *Streptomyces* spp.

In order to trace the transfer of pI, the plasmid was labeled by integration of an apramycin resistance cassette via PCR-targeted gene deletion as described above. The cassette was inserted into a 450-bp gene coding for a putative PadR family transcriptional regulator (locus tag, SMIR_04970) resulting in p-apr that had been established as a locus for gene integration in *Streptomyces* genetics (77).

Plasmid transfer from S. mirabilis P16B-1 to S. lividans TK24 was tested on plates and in sterile soil microcosms. For plate matings, spores of both strains were plated on CSA or MS agar containing 10 mM MgCl_2_. After 7 days of incubation, the plates were overlaid with soft agar (12.5 g/L nutrient broth and 7 g/L agar) and amended with 25 μg/mL apramycin and 12 μg/mL chloramphenicol for selection. After four additional days of incubation, transconjugants were isolated and plasmid transfer confirmed by PCR and Southern blot analysis targeting the resistance cassette.

Soil samples for sterile soil microcosms were taken in Paradiespark, Jena, Germany (for detailed soil characterization, see reference 4), dried at 60°C, and sieved (2 mm grain size). Seventeen grams of soil in 50-mL tubes (Greiner Bio-One) were autoclaved, humidity adjusted with sterile distilled water to 80%, and the microcosms inoculated with spore suspensions of donor and recipient in three replicates. The microcosms were incubated at 28°C in the dark. Reisolation was carried out after 6 days by removing the upper 0.5-cm soil layer and resuspending it in 2 mL saline solution. For transconjugant selection, a dilution series was plated on selective agar.

### Heterologous expression in E. coli.

The impact of streptomycete genes on growth of E. coli was screened by transforming E. coli TransforMax with either pTrc99A, pSEThph, or a derivative carrying streptomycete genomic DNA. The assays were conducted in 96-well cell culture plates filled with 200 μL LB (amended with the appropriate antibiotics and metal salts) and 30 μL E. coli culture (OD_595_, 0.15) per well in six replicates. When using pTrc99A and its derivatives, 1.5 mM isopropyl β-d-1-thiogalactopyranoside (IPTG) was added. The plates were incubated at 37°C, and growth was monitored at *A*_595_ every hour for 24 h using a VERSAmax microplate reader (Molecular Devices, San Jose, CA, USA). Growth curves were compared for significant differences using a CGGC permutation test with 1,000 permutations (78).

### Data availability.

The genome sequence has been published with NCBI GenBank (accession no. CP074102 for the chromosome and accession no. CP074103 for the plasmid sequences).
