# Supplementary material for: Extremophile Metal Resistance: Plasmid-Encoded Functions in Streptomyces mirabilis
Source: Appl Environ Microbiol. 2022 May 23;88(11):e00085-22. doi: 10.1128/aem.00085-22 (PMC9195940; doi:10.1128/aem.00085-22)
Supplement: Supplemental file 1 — Fig. S1 to S9 and Tables S1 to S5. Download aem.00085-22-s0001.pdf, PDF file, 0.7 MB [file aem.00085-22-s0001.pdf]

Supplemental Material

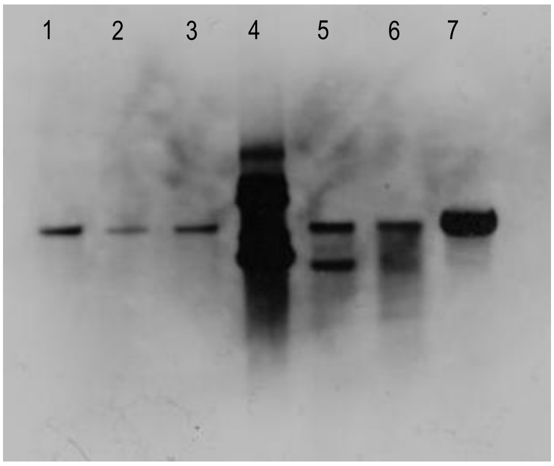

Suppl. Fig. S1. Southern blot analysis verifying successful transformation by using a probe directed against the introduced apramycin resistance cassette. *Bam*HI-digested DNA from six *S. mirabilis* P16 transformants (1-3, 5-7) was checked using *aac(3)IV* encoded on pSET152 as a probe. As control (4) the plasmid pSET152 carrying the apramycin cassette was used.

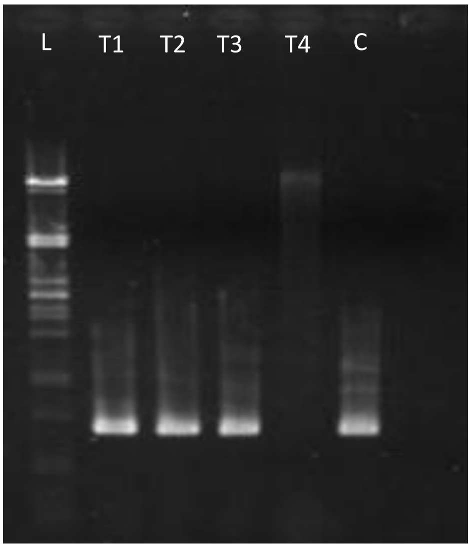

Suppl. Fig. S2. Verification of plasmid transfer into *Streptomyces lividans* TK24 transconjugants. PCR using primers specific for plasmid sequences verified plasmid transfer in three out of the four tested exconjugant strains (T1, T2, T3). PstI-digested lamda DNA served as ladder (L) and *S. mirabilis* P16B-1 DNA as positive control (C).

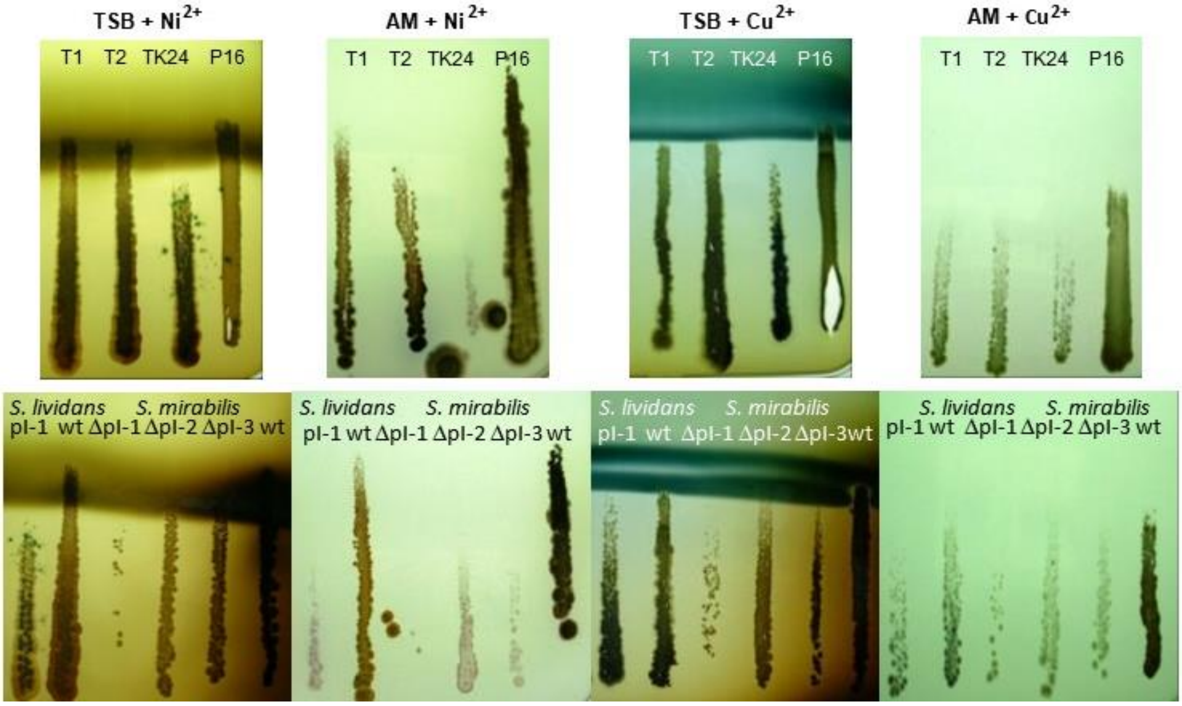

Suppl. Fig. S3. Trench plate test for metal tolerance using *S. lividans* transconjugants (T1, T2) carrying the plasmid compared to the donor *S. mirabilis* P16B-1. Nickel and copper tolerance were compared in plates with complex TSB or minimal AM medium, in which a trench was cut at the top. Growth towards a trench containing the metal salt solution (at the top) was tested to produce a metal concentration gradient starting from the trench; sensitivity is seen by lack of growth towards the trench. The transfer of pI increased the metal tolerance of the recipient *S. lividans* TK24 (upper panel). Three cured strains of the donor *S. mirabilis* P16B-1 (ΔpI-1, ΔpI-2, ΔpI-3) were included in this test (lower panel).

Suppl. Tab. S1. Classification of biomineral formation on TSB agar plates containing at least 1 mM nickel for the wildtype strains *S. mirabilis* P16B-1 and *S. lividans* TK24, as well as the plasmid-free *S. mirabilis* ΔpI and the plasmid-bearing transconjugant *S. lividans* pI. The test was repeated at different concentrations and always with n>3. To compare different tests, a classification system was applied, where the highest observed amount of crystals was set to the highest class, 5; 0, no minerals found (for visualization compare Fig. 2 in main text; partly visible also in suppl. Fig. S3).

	crystal formation	classification	description
S. mirabilis P16B-1	number	2	consistently low numbers, but large crystals
	size	5	
S. mirabilis ΔpI	number	0-1	rarely observed, if so, very small
	size	1	
S. lividans TK24	number	0-3	inconsistent, if crystals were formed, they were very small to large in one experiment, and in medium numbers
	size	1 + 5	
S. lividans pI	number	5	very consistent through all experiments, always in high numbers and very large size
	size	5	

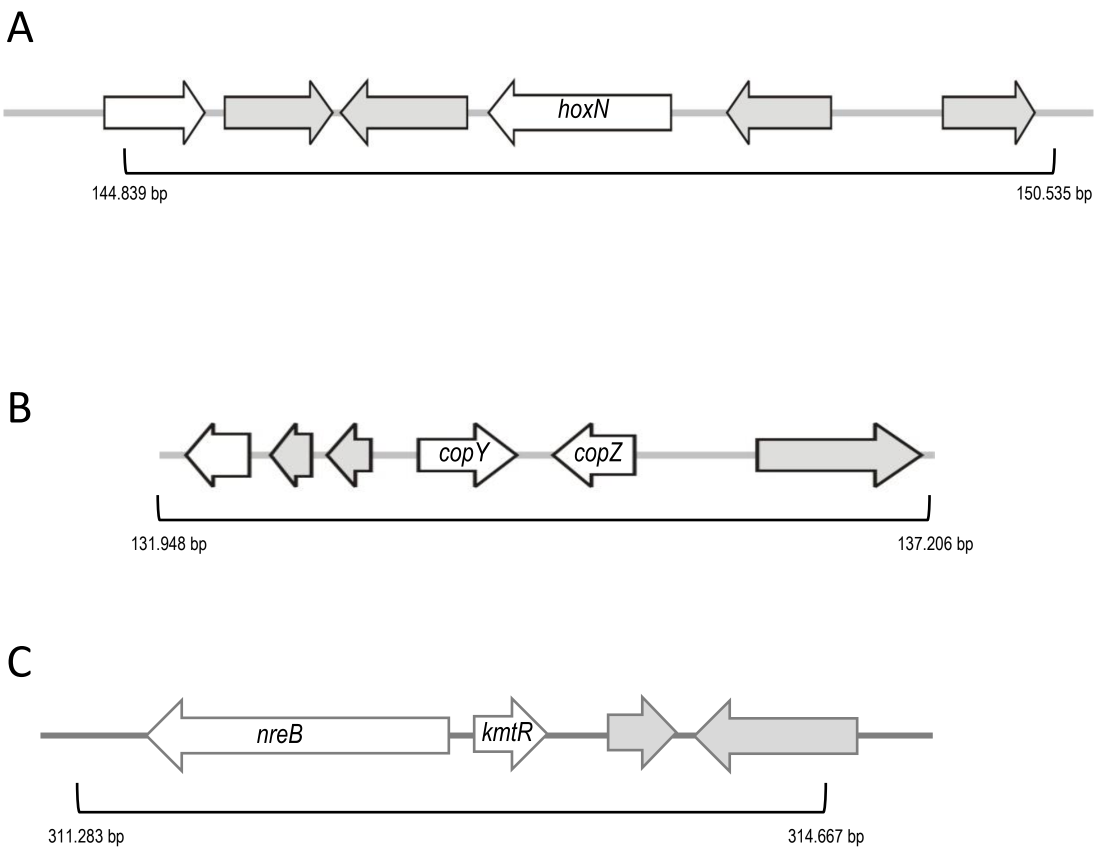

Suppl. Fig. S4. Schematic to-scale representation of the genetic region on the plasmid of *S. mirabilis* P16B-1 coding for the putative nickel transporter HoxN (A), the copper-dependent transcriptional regulator CopY and copper chaperone CopZ (B), and the putative nickel efflux pump NreB with transcriptional regulator gene, *kmtR* (C). Putative open reading frames of unknown function are shaded in grey and the bracket indicates the fragment used for cosmid construction and complementation.

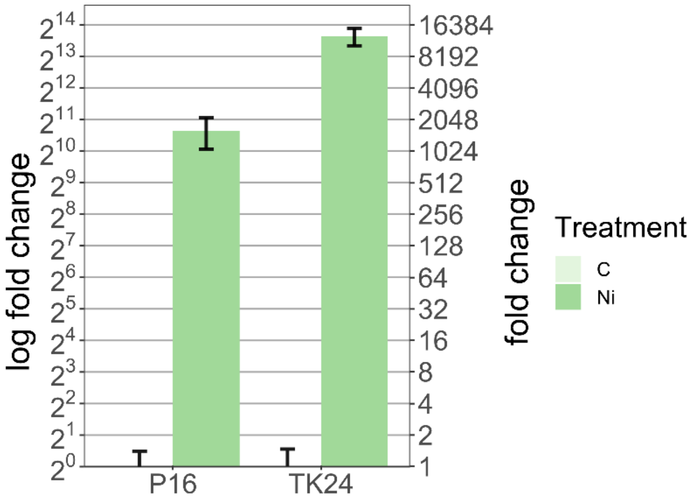

Suppl. Fig. S5. Fold change of *nreB* expression. Cells were grown in 100 ml TSB medium in two treatments, control (C) and 10 mM $NiSO_4$ (Ni) amendment (n=3). With the reference genes, it was possible to establish relative expression in the nickel treatments of both strains. There, *S. lividans* TK24 expressed *nreB* 9,71-fold higher than *S. mirabilis* P16B-1.

Suppl. Tab. S2. Plasmid-encoded *S. mirabilis* p16B-1 genes with changes in RNA level at least 2-fold upon addition of 5 mM Ni to AM medium. Genes investigated in this work are annotated with their gene names. Adjusted p-values (B/H adjustment: padj) were used after multiple testing; NA identifies genes where one of the conditions did not lead to sufficient reads for statistical comparison of all conditons and replicates; (n=3).

Gene identifier	Putative gene function	log2-fold change	padj
	**Up-regulated**		
SMIR_42025	*nreB*	4.12 (17.39-fold up-regulated)	NA
SMIR_42035	hypothetical	3.39	0.02
SMIR_42050	hypothetical	2.63	0.10
SMIR_42040	hypothetical	2.42	0.19
SMIR_41160	*copZ*	2.08 (4.23-fold up-regulated)	NA
SMIR_41740	hypothetical	1.84	NA
SMIR_40585	conjugal transfer protein	1.65	1.00
SMIR_41365	response regulator transcription factor	1.41	1.00
SMIR_41705	hypothetical	1.20	NA
SMIR_42030	*ktmR*	1.09	NA
	**Down-regulated**		
SMIR_40615	glycyl-glycine endopeptidase ALE-1 precursor	-1.12	NA
SMIR_40590	hypothetical	-1.13	1.00
SMIR_40900	GTP binding protein	-1.15	0.84
SMIR_40660	hypothetical	-1.16	1.00
SMIR_41790	pilus assembly protein	-1.23	1.00
SMIR_42365	hypothetical	-1.36	NA
SMIR_40650	hypothetical	-1.53	1.00
SMIR_41715	hypothetical	-1.54	1.00
SMIR_41795	hypothetical	-1.65	NA
SMIR_41540	putative L,D-transpeptidase LppS precursor	-1.81	1.00
SMIR_41710	hypothetical	-2.04	NA
SMIR_40895	methyltransferase domain containing	-2.64	NA
SMIR_41220	*hoxN*	-2.65 (6.28-fold down-regulated)	0.73
SMIR_42450	cation transporter	-3.07	NA

Suppl. Tab. S3. Plasmid-encoded *S. mirabilis* p16B-1 genes with changes in protein level at least 2-fold upon addition of 5 mM Ni to AM medium. Peptides for genes investigated in this work were not detected albeit for nreB, which was present only in the runs with nickel, but absent without nickel (first line; n=2).

Gene identifier	Gene function	Change Ni vs. control	log2-fold change
	**Up-regulated**		
SMIR_42025	*nreB*; protein run 1 peptide count + Ni: 4.12 x 10^8; protein run 2 peptide count + Ni: 9.98 x 10^8; - Ni not detected		
SMIR_42645	α-KG-dep. 2,4-dichlorophenoxyacetate dioxygenase	28.3849	4.83
SMIR_41865	hypothetical	17.2118	4.11
SMIR_41840	hypothetical	14.2873	3.84
SMIR_42210	cytochrome P450	14.0930	3.82
SMIR_40565	HTH-type transcriptional repressor YtrA	10.3693	3.37
SMIR_41970	YncE family protein	6.2097	2.63
SMIR_42990	recombination protein F	4.2849	2.10
SMIR_41990	dihydrolipoamide dehydrogenase	4.1858	2.07
SMIR_41745	hypothetical	3.5082	1.81
SMIR_43055	hypothetical	3.1055	1.63
SMIR_42505	putative amino acid permease YhdG	2.9892	1.58
SMIR_41785	TadE-like	2.9192	1.55
SMIR_41085	integral membrane protein, YkoY family	2.8216	1.50
SMIR_42490	dihydropyrimidine dehydrogenase subunit A	2.7771	1.47
SMIR_41755"	flagella basal body P-ring formation protein FlgA	2.6582	1.41
SMIR_42110	HTH domain containing protein	2.5825	1.37
SMIR_41480	Pspa/IM30 family protein	2.3870	1.26
SMIR_42270	SAM dependent methyltransferase	2.1962	1.14
SMIR_41595	transcriptional regulator CopY	2.1281	1.09
	**Down-regulated**		
SMIR_41905	helix-turn-helix protein	0.3951	-1.34
SMIR_41255	putative periplasmic or secreted lipoprotein	0.3555	-1.49
SMIR_40600	type IV secretory pathway, VirB4 components	0.3344	-1.58
SMIR_41495	HflK protein	0.3307	-1.60
SMIR_40960	copper-transporting P-type ATPase	0.2890	-1.79
SMIR_41030	put. protein-S-isoprenylcysteine methyltransferase	0.2598	-1.94
SMIR_41625	put. protein-S-isoprenylcysteine methyltransferase	0.2598	-1.94
SMIR_40985	oleate hydratase	0.2099	-2.25

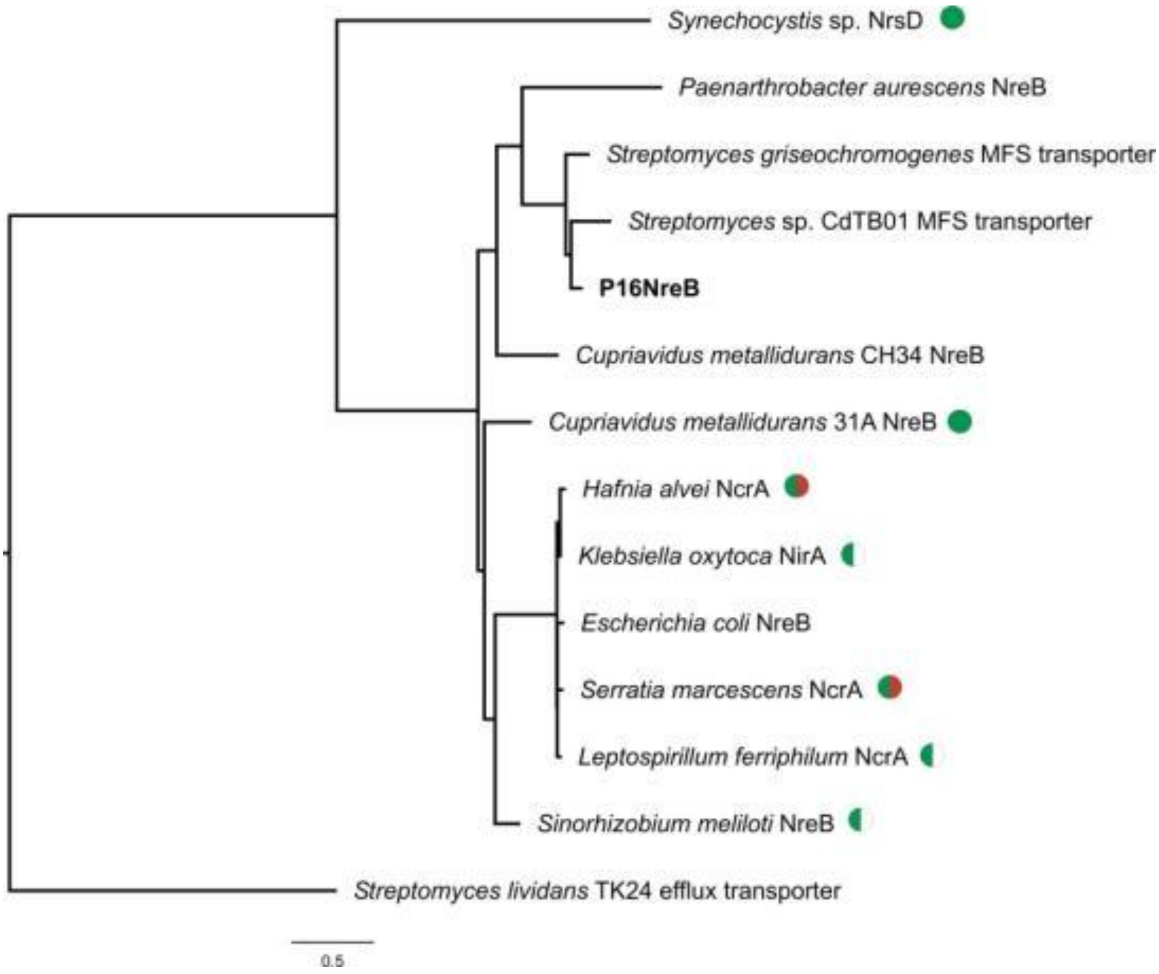

Suppl. Fig. S6. Phylogenetic tree of NreB encoded on the *S. mirabilis* P16B-1 plasmid and other transporters of this family. Some of these transporters were annotated with reference to a functional characterization. This is indicated with full green circles for transport of exclusively nickel, green and brown circles indicate transport of both, nickel and cobalt, and a green half-circle was used to indicate that only nickel transport had been tested for the respective efflux transporter.

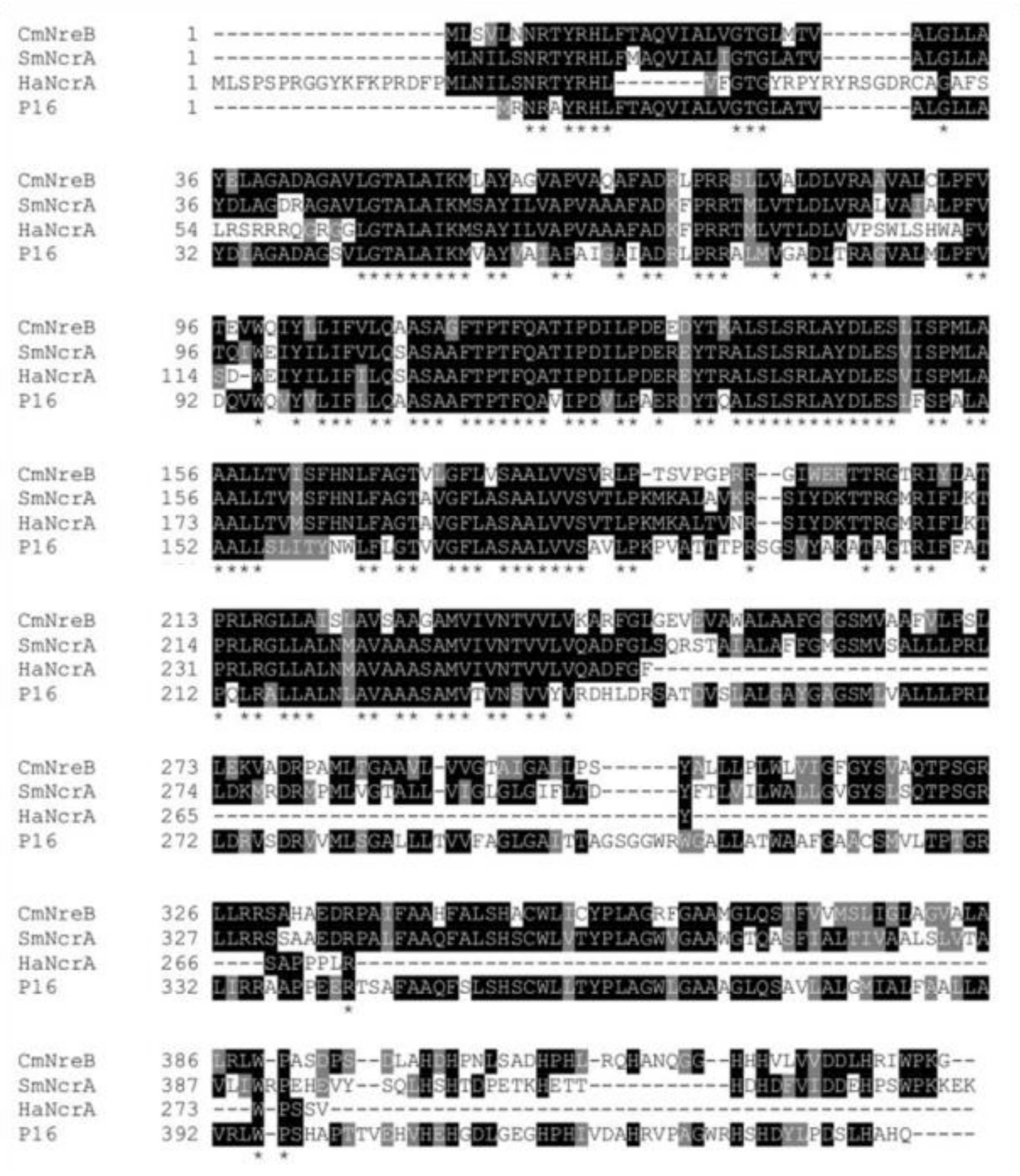

Suppl. Fig. S7: Amino acid sequence alignment of NreB homologs comparing NreB of *S. mirabilis* P16B-1 to *Cupriavidus metallidurans* NreB (ABF13003.1), *Serratia marcescens* NcrA (ABF14406.1) and *Hafnia alvei* NcrA (AAL37245.1). Identical residues are shaded in black, conservative amino acid exchanges in grey.

Suppl. Tab. S4: Maximally tolerated H_2O_2.The addition of induced oxidative stress that limited growth at the given concentration.

	H_2O_2 [ppm]
S. mirabilis P16B-1	30 *
S. mirabilis ΔcopY	39
S. mirabilis ΔcopZ	21
S. lividans TK24 empty vector control	30
S. lividans CopZ	12
S. mirabilis Δpl	30
S. mirabilis Δpl; cosmid containing copYZ	30
S. lividans TK24	30
S. lividans cosmid containing copYZ	12

* growth evaluated after 5 d

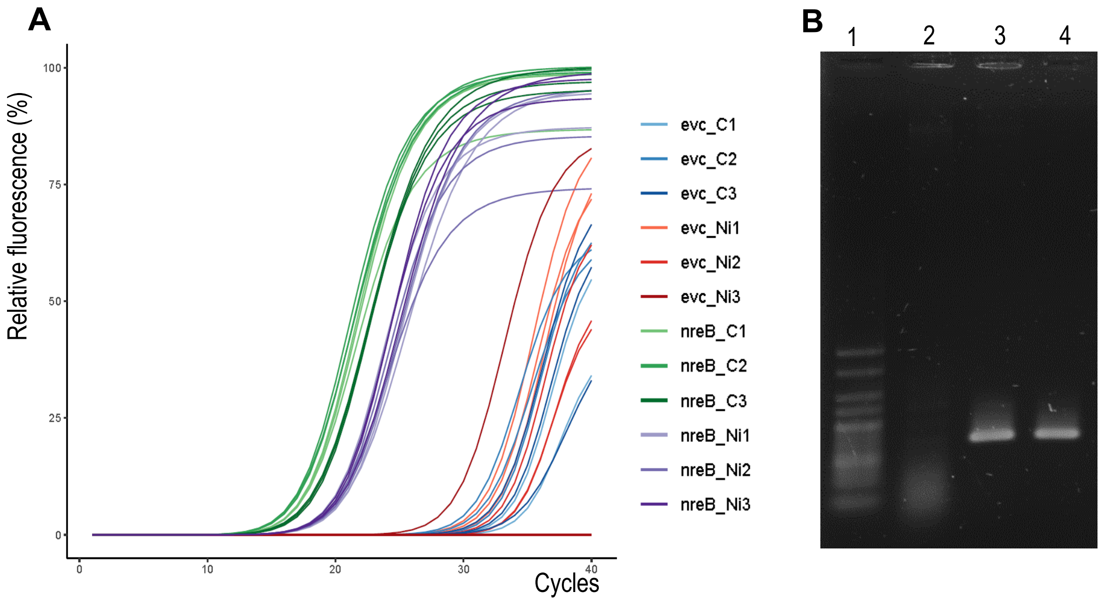

Suppl. Fig. S8: Control for cloning and expression of *E. coli* carrying the empty vector control (evc) or the construct. (A) PCR showing that the vector pTRC99C contained the insert using the same primers and conditions also given for cloning in *S. mirabilis*; hyperladder V (lane 1) and DNA from E. coli transformants with the empty vector control (2) or the construct carrying *nreB* (3), and *S. mirabilis* wildtype for positive control (4) was used as template. (B) To show expression in the heterologous host, qRT-PCR was performed as described for *S. mirabilis* from total RNA of transformants carrying the empty vector control (evc) or the construct (nreB) after growing three biological replicates with or without 1.25 mM $NiSO_4$.

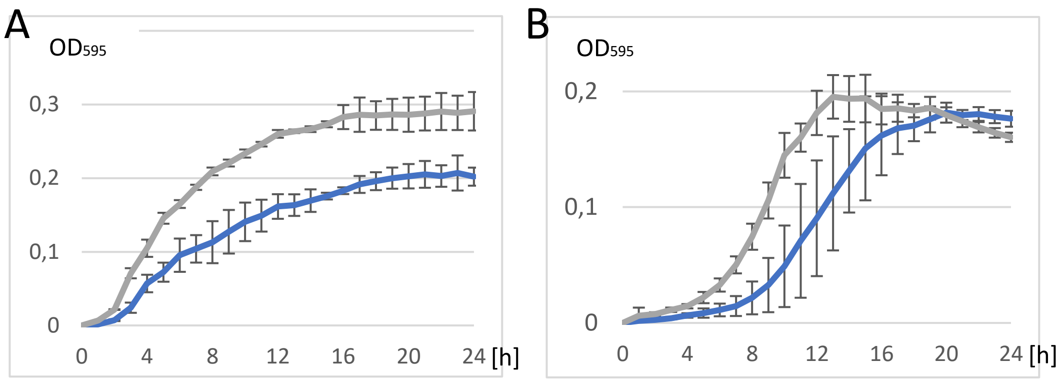

Suppl. Fig. S9: Growth of *E. coli* transformants carrying the empty vector control (blue) or the construct with 3.4 kb genomic region carrying *nreB* (grey) in LB medium supplemented with (A) 1.25 mM $NiSO_4$, or (B) 0.75 mM $CoSO_4$; bars indicate standard deviation of six replicates. The growth was assayed in 96 well cell culture plates after induction with 1.5 mM isopropyl β-D-1-thiogalactopyranoside (IPTG) at 37°C (for the cloned region containing *nreB* and *kmtR*, see Fig. 4).

Suppl. Tab. S5: Results of significance test comparing all data points in a between growth curves of *E. coli* transformed with *nreB* and control strains at different salt levels. Growth was measured in ELISA plates and curves compared for significant differences using a CGGC permutation test with 1000 permutations (see Materials and Methods).

Salt	concentration	Stat (observed value of the statistic)	P value	Significance at α=0,01	Significance at α=0,05
$NiSO_4$	2 mM	-11,9	0,002	yes	yes
	2,5 mM	-2,08	0,02	no	yes
$NiCl_2$	1,5 mM	-11,8	0,003	yes	yes
	2 mM	-23,4	0,004	yes	yes
$CoSO_4$	0,5 mM	-3,68	0,003	yes	yes
	1 mM	-1,52	0,059	no	no
$CuSO_4$	2,5 mM	-2,55	0,004	yes	yes
	3 mM	-4,31	0,002	yes	yes
